# Giant Condyloma Acuminatum of Vulva Frustrating Treatment Challenge

**Published:** 2015-07

**Authors:** Feizollah Niazy, Khalil Rostami, Amir Reza Motabar

**Affiliations:** Department of Plastic Surgery, Shahid Beheshti University of Medical Sciences, Modaress Hospital, Tehran, Iran

**Keywords:** Giant condylomata, Buschke-Löwenstein tumour, Reconstruction, Vulva

## Abstract

Giant condylomata are not usually seen nowadays in developed nations, but such cases are still seen in the under-resourced countries. Condylomata acuminata are commonly transmitted through sexual intercourse. Generally diagnosed based on their appearance. Giant condyloma acuminata also named Buschke- Löwenstein tumour (BLT) is a slow growing cauliflower-like tumor, locally aggressive and destructive, with possible malignant transformation. Common clinical treatment of anogenital warts is conservative, however, in extreme cases conservative therapy is insufficient and surgical excision is required. A case of common presentation of giant condylomata in a 50 years old, divorced, multiparous woman is presented and the literature is reviewed. She presented with 15 years history of slowly progressive vulval lesion and associated itching, contact bleeding, malodorous vaginal discharge and difficulty in walking. She had previously been treated with podophyllin and cryosurgery without success. The growth measured 30×10 cm in each side and was successfully excised with no evidence of malignancy concomitant and reconstruction also done.

## INTRODUCTION

Anogenital warts (condyloma acuminatum or venereal warts) are a common sexually transmitted disease among females and males.^1^^,^^2^ The causal role of human papillomaviruses (HPV) in anogenital wart formation has been firmly established biologically and epidemiologically.^3^^-^^5^ Genital HPV infections are transmitted primarily through sexual contact, with a lifetime risk of 50-80%.^6^ The highest rate of genital HPV infection has been identified in adults between 18 and 28 years of age.^7^^,^^8^ Although 90% of those who contract HPV will not develop genital warts, those infected can still transmit the virus. The immune system effectively repels the majority of HPV infections and is associated with marked localized cell mediated immune responses. However, approximately 10% of individuals develop a persistent infection, with risk of developing benign proliferative lesions, high-grade precursors and eventually invasive carcinomas.^9^ HPVs are classified into high- or low-risk types depending on oncogenic potential. Low-risk types 6 and 11 are isolated in approximately 90% of genital wart cases.^3^ The most common clinical treatment is conservative, with local chemical or physical destruction and immunological therapy.^10^ In more extreme cases conservative therapy is insufficient and surgical excision is required.

Giant condyloma acuminata (GCA; Buschke-Löwenstein tumour) is an extremely rare clinical form of genital warts, characterized by aggressive down growth into underlying dermal structures.^11^^,^^12^ A complex histological pattern may exist with areas of benign condyloma intermixed with foci of atypical epithelial cells or well differentiated squamous cell carcinoma. GCA is mainly localized to the genital region, however, in rare cases the tumor is localized to distinct histological zones of the anorectal region. Due to infiltration of the underlying tissue, fistulae and abscesses may be observed. GCA is resistant to chemotherapy or radiotherapy and usually requires local radical resection for curative treatment. We report a case of 50-year-old lady with GCA of vulva who was successfully treated by simple vulvectomy and reconstruction of the defect by ‘‘two-advanment flap technique’’.

## CASE REPORT

**Fig. 1:**
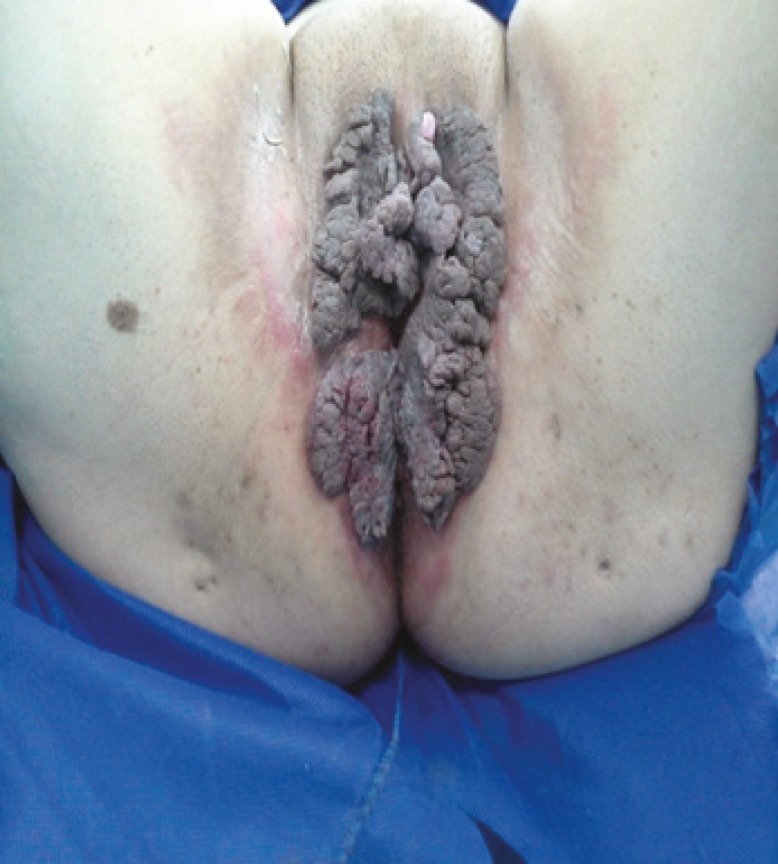
Patient with giant condyloma prior to reconstructive surgery

A 50-year-old female presented with cauliflower-like growth over the uvula region. The growth had been diagnosed previously as condyloma acuminatum which was resistant to conservative therapy (Figure 1). The patient was 35-year-old when she first noted the lesion and did not seek any medical help. Two years later she became pregnant and during pregnancy period, she received conservative treatment and lesion disappeared after her delivery, After 2 years during the first trimester of the patient’s second pregnancy, warts appeared for the second time with altered clinical presentation; spread across the entire uvula region she underwent cryosurgery, but with no significant improvement and she did not any regular follow up so far. 

On examination, there was a huge friable, foul smelling growth on her vulva involving both labia majora, extending anteriorly to mons pubis and posteriorly to perianal region (Figure 1). There were a few satellite lesions on labia minora and perianal region. Clitoris, urethra and external anal sphincter were not involved. Colposcopic, proctoscopic and oropharyngeal examinations were normal. The patient’s medical history also was negative. The patient was screened negative for sexually transmitted diseases and high-risk HPV DNA.

**Fig. 2:**
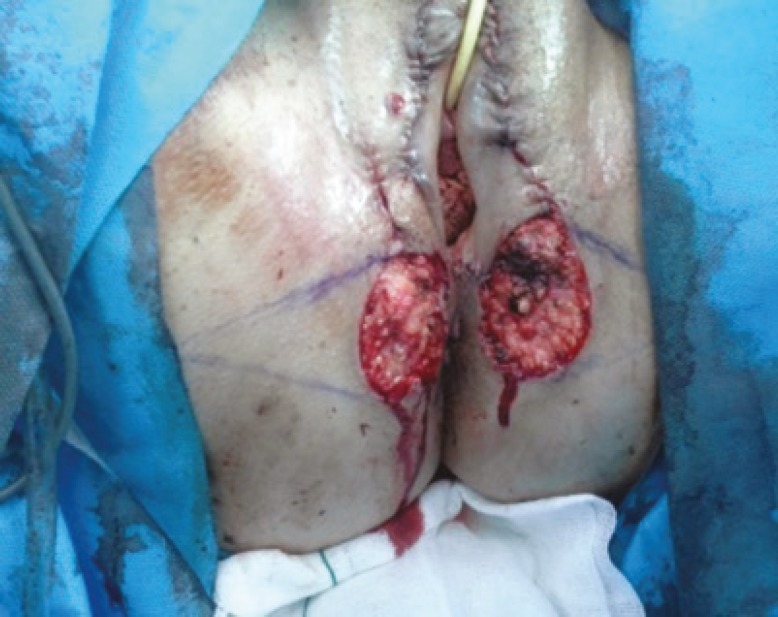
Patient during the surgical procedure

**Fig. 3:**
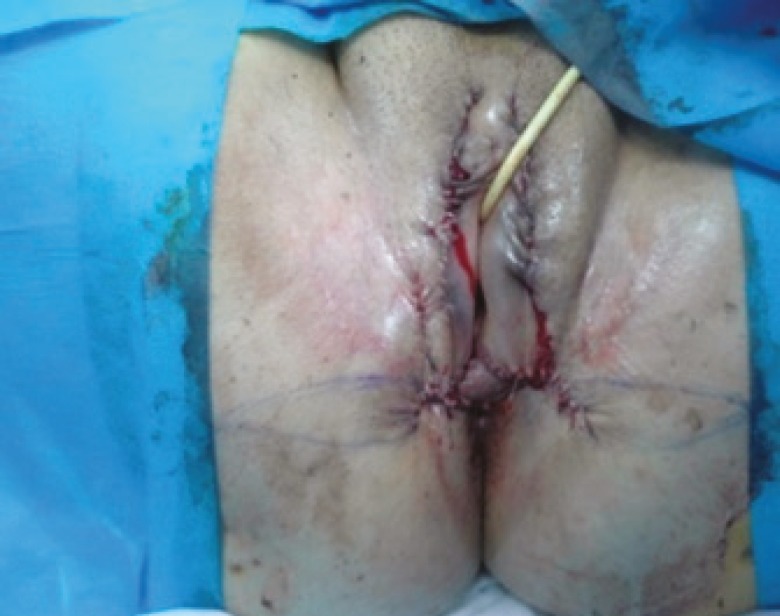
Patient after surgery.

A simple vulvectomy, achieving wide margins, was performed. Small lesions in perianal area and labia minora were also excised. Reconstruction of vulva was performed using ‘two flap advancement technique’. In this technique, two random pattern flaps in the dimension of 1:1 or 1:2 were raised adjacent to the defect and advanced medially to resurface the defect. This resulted in defect being transferred laterally, but this defect was smaller than the original defect and could be closed primarily (Figure 2 and 3).

**Fig. 4:**
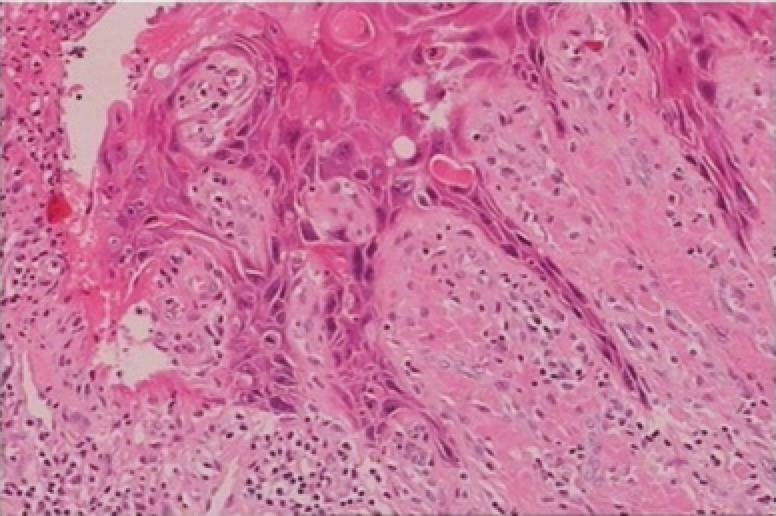
Histology of the giant condyloma

**Fig. 5:**
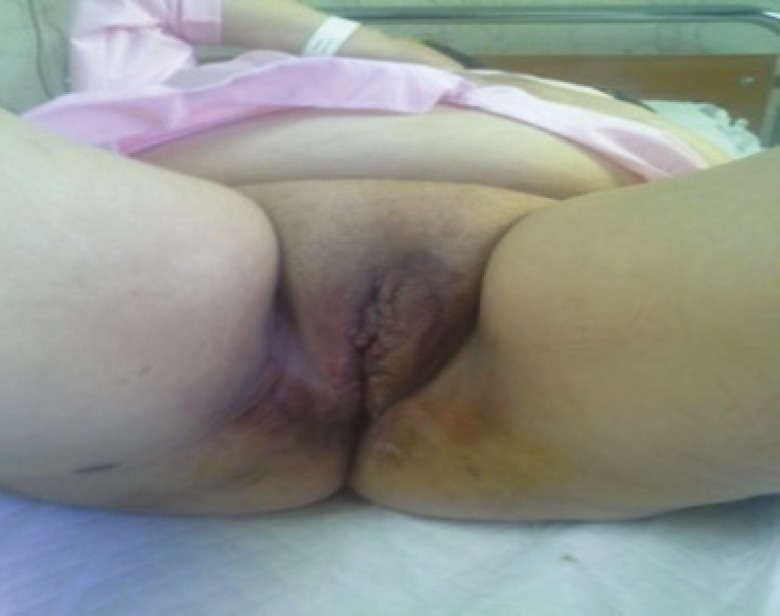
Patient following the healing process

Final histopathology report confirmed the diagnosis of condyloma acuminatum with mild degree of dysplasia (Figure 4). Functional outcome was good with no sexual complaints. She remained free of recurrence at 12 months of follow-up (Figure 5).

## DISCUSSION

Anogenital warts are the most common outcome of HPV genital infection. Therapeutics against this sexually transmitted disease are currently associated with low efficacy, due to a 30-70% recurrence rate identified six months following therapy administration.^10^ In rare cases, anogenital warts develop into extremely large tumor masses leading to deterioration of patient quality of life. An identified underlying histopathology of specific cases of giant condyloma is superficial planocellular carcinoma. 

Patients with high susceptibility to local development and fast progression (in growth and malignancy) and the highest rate of recurrence often exhibit various types of immunodeficiency. In addition, immunodeficiency leads to difficulties in evaluation of optimal therapeutic management. However, patients with no marked immunodeficiency and treatment-resistant genital warts have been identified. Furthermore, condyloma lesions occasionally form large exophytic masses, interfering with intercourse, normal urination, defecation or vaginal delivery.

Commonly, GCA develops as cauliflower-like masses and the tumors exhibit histological features of pseudo-epitheliomatous proliferation and local invasion. In the absence of metastases, they are termed Buschke-Löwenstein tumors. Due to the aggressive local development of these masses, they belong to the verrucous carcinoma group, although a malignant histological alteration in the form of micro-invasive carcinoma or well-differentiated epidermoid keratinizing carcinoma has been reported. Due to the high frequency of local recurrence, radical surgical excision is the current treatment of choice as topical preparations and chemotherapy are generally considered ineffective.^13^ The method selected for reconstruction is crucial, particularly in neglected cases similar to the present case study. Local tissue availability and the patient’s condition and attitude towards the development of the disease are major factors for reconstruction with local fascio-cutaneous flaps. Five years following surgery, the present patient is disease-free with no recurrence.

Many treatment strategies have been documented in the literature for management of GCA, but mainly in the form of case reports. Complete surgical excision with histologically clear margins, with or without adjuvants, is the mainstay of the treatment for GCA, including that of vulva.^1^^,^^3^ Other modalities include combined radiotherapy and chemotherapy, topical agents (e.g. podophyllin, 5-FU, imiquimod, bleomycin) and intralesional injections of interferons.^4^^-^^7^

The skin defects created after excision of GCA in the peri-anal region can be managed with mesh skin grafting, flaps and even healing by secondary intention.^1^^,^^8^^-^^10^ Flaps have been shown to give better results for reconstruction of vulva.^9^^,^^10^ The additional advantage of our ‘four flap technique is that the scars do not extend over the perineum or thighs. In our patient, the postoperative period was smooth. The healing duration was shorter (3 weeks) and scarring was also less as compared to that described after mesh skin grafting.^8^ To conclude, surgical treatment using wide local excision of the lesion with clear margins and reconstruction of the resultant skin defects by two transpose flap technique gives excellent cosmetic and functional results.

## CONFLICT OF INTEREST

The authors declare no conflict of interest.

## Note

Please cite this paper as:

Niazy F, Rostami K, Motabar AR. Giant Condyloma Acuminatum of Vulva Frustrating Treatment Challenge. World J Plast Surg 2015;4(2):159-162.
